# Resistance Training and Handball Players’ Isokinetic, Isometric and Maximal Strength, Muscle Power and Throwing Ball Velocity: A Systematic Review and Meta-Analysis

**DOI:** 10.3390/ijerph17082663

**Published:** 2020-04-13

**Authors:** Nicola Luigi Bragazzi, Mehdi Rouissi, Souhail Hermassi, Karim Chamari

**Affiliations:** 1 Department of Mathematics and Statistics, Laboratory for Industrial and Applied Mathematics (LIAM), York University, Toronto, ON M3J 1P3, Canada; 2 Tunisian Research Laboratory, National Centre of Medicine and Science in Sport, Tunis 1003, Tunisia; mehdirwissi@gmail.com; 3 Sport Science Program, College of Arts and Sciences, Qatar University, Doha 2713, Qatar; 4 ASPETAR, Orthopedic and Sports Medicine Hospital, Doha 29222, Qatar; karim.chamari@aspetar.com; 5 Laboratory “Sport Performance Optimization”, National Center of Sports Medicine and Sports Sciences (CNMSS), ISSEP Ksar-Said, Manouba University, Tunis 2010, Tunisia

**Keywords:** strength training, peak power, team handball, systematic review, meta-analysis

## Abstract

Purpose: Handball (Team Handball) is an intermittent and strenuous contact sport, the successful performance of which depends on frequent body contacts, and the ability to make repeated explosive muscular contractions required for jumping, acceleration, sprinting, turning, changing pace, and throwing a ball. Many studies have investigated the effect of resistance training (RT) in handball players, however with conflicting results. Therefore, our objective was to investigate the impact of RT on maximal strength (isometric and isokinetic strength), the power of both lower and upper limbs, and throwing velocity, in handball players. Methods: A comprehensive literature search yielded a pool of 18 studies, which were retained in the systematic review and meta-analysis. Results: A total of 275 handball players were included. The overall effect size (ES) of RT was 0.996 ([95%CI 0.827–1.165], *p* = 0.0000). At the multivariate meta-regression, the effect of publication year was significant, as well as the effects of country, gender, and level. The impact of RT on isokinetic strength was not significant (ES 0.079 [95%CI −0.060–0.219], *p* = 0.265), whereas the impact of RT on throwing (ES 1.360 [95%CI 0.992–1.728], *p* = 0.000) was significant, as well as the effects of RT on isometric strength (ES 0.398 [95%CI 0.096–0.700], *p* = 0.010), on maximal strength (ES 1.824 [95%CI 1.305–2.343], *p* = 0.000), and on power (ES 0.892 [95%CI 0.656–1.128], *p* = 0.000). Conclusions: RT has a significant impact in handball players. Handball coaches could design conditioning protocols and programs based on our results. However, due to a number of shortcomings, including the high, statistically significant heterogeneity among studies and the evidence of publication bias, further high-quality investigations are needed.

## 1. Introduction

Team handball is a sport that requires physical efforts of high-intensity and of short duration with a strenuous contact and a particular ability to make and repeat explosive muscular contractions. During a handball match, more than 825 high intensity actions can be performed, requiring a high level of strength [1,2,3,4]. Maximal strength, power and throwing velocity are considered as major determinants of success in elite handball players. Hence, handball coaches should perform specific handball conditioning, including high intensity exercises such as resistance training (RT) to develop these physical qualities. RT involves the use of wide range of resistive loads and a variety of training modalities (i.e., throwing with regular balls, throwing with a medicine ball, Olympic weight lifting, elastic band training and plyometric training) aimed at developing maximal strength and/or muscular power.

Throwing is a fundamental skill in handball. Two basic factors influence the efficiency of shots: accuracy and throwing ball velocity. The faster the ball is thrown, the less time defenders and/or goal keeper have to save the shot. Coaches and scientists seem to have reached an agreement stating that the main determinants of throwing ball velocity are the timing of movement in consecutive body segments, the technique used, and the strength and power of both the upper and lower limbs [1,2,3]. Each of these factors can be improved by training, particularly RT designed to enhance strength and power in both the upper and lower limbs. However, training programs that produce the greatest change in muscle cross-section typically involve loads of 70% with one repetition maximum (1RM) [2], whereas programs designed to improve strength through enhanced neuronal coordination are characterized by intensities of 85–100% 1RM [2].

Many studies have investigated the effect of RT on throwing velocity in handball players, but with conflicting results. However, differences in the intensity of training may also have contributed to conflicting results. For instance, Chelly et al. [5] indicated that upper limb plyometric training improved throwing ball performance in the RT trained group, with no significant improvement in the control group, whereas, for instance, other studies noted a significant enhancement (*p* < 0.001) in standing handball throwing velocity after 6 weeks of heavy upper limb RT. Furthermore, Hermassi et al. [6,7,8] reported similar results after RT programs. On the other hand, Raeder et al. [9] reported a significant increase in throwing velocity of 14% and 3.7% for both training and control groups, respectively. Genevois et al. [10] indicated non-significant improvements in throwing ball velocity for the dominant arm following 6 weeks of a strengthening program.

Moreover, the major role of the lower limb strength and power has also been highlighted in many studies demonstrating a significant relationship between such high intensity actions and lower limb strength/power [11,12,13]. To maintain a high level of strength, both strength/power training should be carefully monitored throughout the competitive season. RT programs could be very useful to develop lower/upper limb strength and to maintain it throughout the season, both for male and female handball players [11,12,13]. Specific handball studies on this issue have examined the impact of speed strength programs on muscular power, jump performance or throwing velocity [5,11,12,13,14,15]. Gorostiaga et al. [15] showed that specific RT can improve the strength of both the upper extremity muscles (23%; *p* < 0.01) and the leg extensors (12.2%; *p* < 0.01), but no gains can be anticipated from the low resistance forms of activity involved in team handball practice. Marques and Gonzalez-Badillo [14] also noted a 28% increase in 1RM bench press in high level handball players after 12 weeks of RT (2–3 sessions per week), close to the 34% gain in 1RM bench press reported by other studies [6]. Their loadings were in the range 70–85% of concentric 1RM bench press. Hermassi et al. [6] also found upper limb gains of 34% and 20% for pullover and bench press, respectively, over 8 weeks of strength training. This is a larger response than the results previously observed by Gorostiaga et al. [15].

In addition, Gorostiaga et al. [15] are the only previous investigators who have studied the influence of heavy RT on the jump performance of handball players. They reported significant increases in a group that had previously engaged only in team practice (6%; *p* < 0.001), but no changes in counter movement jump (CMJ) for either RT or control groups. Hermassi et al. [6] observed a significant increase in the jump performance of well-trained handball athletes after 8 weeks of heavy squat training, associated with a large improvement in 1RM squat strength.

However, despite the popularity of handball and the importance of RT, no systematic review exists on the topic. Therefore, the objective of this systematic review and meta-analysis was to investigate the effects of RT on maximal strength (isometric and isokinetic strength), the power of both lower and upper limbs and throwing velocity in handball players, hopefully clarifying some of the discrepancies found in the literature. Thus, handball coaches could use this review as a guideline for designing specific RT programs aiming to improve or maintain force-related physical performance of handball players throughout the competitive season and to potentially also reduce the rate of injury.

## 2. Materials and Methods

A systematic review with meta-analysis was performed according to the “Preferred Reporting Items for Systematic reviews and Meta-Analyses” (PRISMA) guidelines [16]. The main review question was: what is the effect of RT on maximal strength, isometric and isokinetic strength, the power of lower and upper limbs and throwing velocity in handball players [17,18]?

In order to answer to this review question, a comprehensive search was carried out, systematically searching the following scholarly electronic databases: namely, PubMed/MEDLINE, the Cochrane Central Register of Controlled Trials (CENTRAL), Scopus and ISI/Web of Science. No limits were placed on publication date (in other words, no time filter was applied: all the scholarly databases were mined from inception). Articles written in any language were eligible. We also manually scanned the reference lists of articles identified by previous electronic searches and looked through the citation analysis in review papers regarding handball to increase the chance of including all relevant studies eventually missed by the original search terms.

The following Participants, Exposure, Comparator, Outcome and Study (PECOS) criteria were followed:

- Participants/population: we included studies where the study population consisted of handball athletes (either amateur or professional players), men or women, youths or adults;
- Exposure(s): we analyzed handball players exposed to RT;
- Comparator(s)/control: we analyzed other kinds of training versus RT;
- Outcome(s): the primary outcome was the effect of the RT on maximal strength, isometric or isokinetic strength, power and/or throwing velocity in handball players.
- Study design: we considered any study design except for studies designed as expert opinion, comment/commentary, editorial/letter to editor, and review.

Gray literature was not considered, since we preferred to focus on high-quality peer-reviewed investigations. Studies that duplicated populations from the same authors were also excluded, as well as studies with a lack of quantitative details or results that were not pertinent to the scope of the present review.

The primary search strategy included the following search terms: handball AND “resistance training” AND (maximal strength OR force OR power OR throwing velocity OR isometric strength OR isokinetic strength). Further details are shown in Table 1.

All data were independently extracted by two reviewers (M.R., N.L.B.) using an ad-hoc designed Excel spreadsheet and independently verified/confirmed by another reviewer, with expertise in the field of handball (K.C.). Firstly, screening of titles and/or abstracts for relevant and possibly eligible citations was performed. Secondly, full text article analysis was conducted to carefully select studies. In the case of disagreements, a consensus was reached through discussion. If disagreements were not resolved, a third reviewer (K.C.) was consulted and acted as final referee.

A meta-analysis has been performed, utilizing the commercial software “Comprehensive Meta-analysis” CMA version 3.0 (Biostat, Englewood, USA). The effect size (ES) was expressed as Hedges’ g, setting a correlation between pre- and post-measures of 0.50, whereas the global ES was computed according to Morris. In cases of considerable heterogeneity (I^2^ > 50%), a random effect model was carried out, instead of a fixed effect model. Evidence of publication bias was verified by both visually inspecting the funnel plot and computing Egger’s linear regression test. The Duval and Tweedie trim-and-fill analysis was performed to compute the true ES, in cases where there was evidence of publication bias. Meta-regression analyses were conducted to shed light on the determinants of the ES.

Results are presented in a tabular form, together with an analysis of the different subgroups or subsets.

## 3. Results

### 3.1. Systematic Review

**Figure 1:**
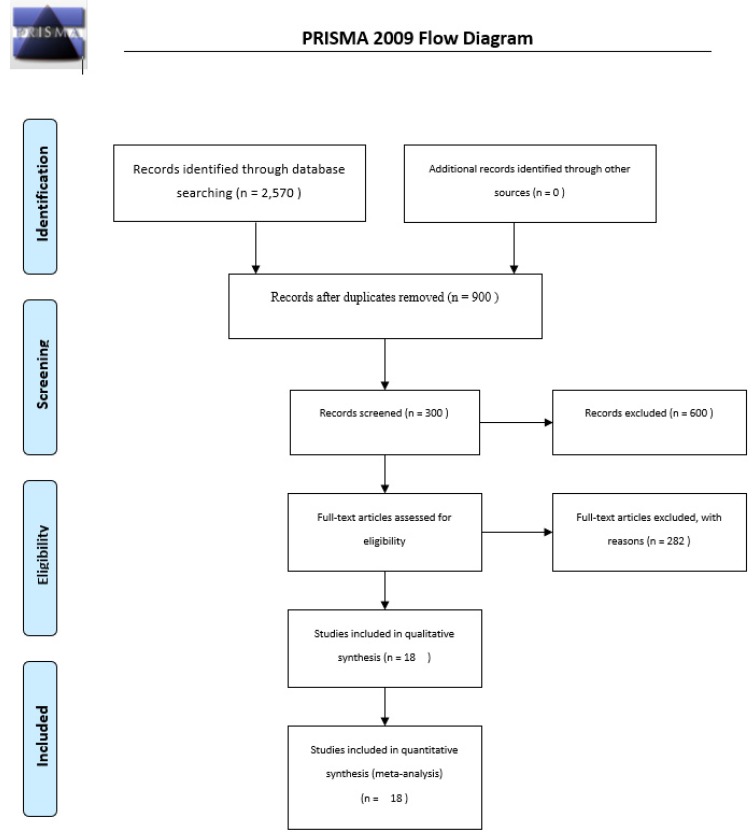
The process of studies retrieval and inclusion adopted in the present systematic review and meta-analysis.

The initial search yielded 2570 studies. Finally, after deleting duplicates and based on inclusion/exclusion criteria, 18 studies [5,6,7,8,9,10,13,14,15,17,19,20,21,22,23,24,25,26] were retained in the present systematic review and meta-analysis (Figure 1). The main features of the studies are presented in Table 2. A total of 275 handball players were included.

### 3.2. Meta-Analysis

The overall ES was 0.996 ([95%CI 0.827−1.165], *p* = 0.0000; k studies = 82). Due to the significant amount of heterogeneity (I^2^ = 82.73%), the random−effect model was carried out. At the multivariate meta−regression (tau^2^ = 0.19, tau = 0.44, I^2^ = 65.55%, Q = 191.57, df = 66, *p* = 0.0000), the effect of publication year was significant (*p* = 0.0005; regression coefficient = 0.0007 [95%CI 0.03−0.11], SE = 0.02, z = 3.46), as well as the effect of country (*p* = 0.0000; regression coefficient = −1.08 [95%CI −1.43 to −0.73] for Europe versus Africa, SE = 0.18, z = −6.06), gender (*p* = 0.0022; regression coefficient = −0.01 [95%CI −0.01 to −0.00], SE = 0.00, z = −3.06), and expertise level (*p* = 0.0346; regression coefficient = 0.92 [95%CI 0.07−1.18] for sub−elite versus elite athletes, SE = 0.44, z = 2.11). The number of weeks was statistically borderline (*p* = 0.0655; regression coefficient = 0.08 [95%CI −0.01 to 0.17], SE = 0.04, z = 1.84), whereas the number of sessions per week was not significant (*p* = 0.4233). The other parameters failed to reach the significance threshold (age *p* = 0.3233). There was a significant difference among the outcome selected (Q = 19.63, df = 4, *p* = 0.0006; for isometric strength, regression coefficient = 0.30 [95%CI −0.40–0.99], SE = 0.35, z = 0.84, *p* = 0.4018; for maximal strength, regression coefficient = 1.11 [95%CI 0.45–1.76], SE = 0.33, z = 3.31, *p* = 0.0009; for power, regression coefficient = 0.30 [95%CI −0.16−0.76], SE = 0.23, z = 3.13, *p* = 0.1994; for throwing, regression coefficient = 0.81 [95%CI 0.30−1.32], SE = 0.26, z = 3.13, *p* = 0.0017). There was evidence of publication bias, both in visually inspecting the Funnel plot and carrying out Egger’s linear regression test (intercept = 5.27 [95%CI 4.74–5.81], SE = 0.27, t = 19.58, *p* = 0.0000). Moreover, with the Duval and Tweedie trim-and-fill analysis, 30 studies were trimmed (thus resulting in a “true ES” of 0.378 [95%CI 0.184–0.571], Q = 928.17).

When stratifying according to expertise level, ES was higher among elite athletes (ES 1.067 [95%CI 0.882–1.251], *p* = 0.000) than among sub−elite athletes (ES 0.510 [95%CI 0.136−0.883], *p* = 0.008; I^2^ = 67.38, random−effect model). Among elite athletes, there was an evidence of publication bias. Among sub−elite athlete studies, there was a small amount of evidence of publication bias (intercept = 7.40 [95%CI 4.99−9.80], t = 7.28, *p* = 0.00017): one study was trimmed, resulting into a “true ES” of 0.350 ([95%CI −0.114−0.813], Q = 43.29).

When stratifying according to gender, ES was lower among female athletes (ES 0.783 ([95%CI 0.435–1.130], *p* = 0.000), than among male athletes (ES 1.042 ([95%CI 0.854−1.230], *p* = 0.000).

**Figure 2:**
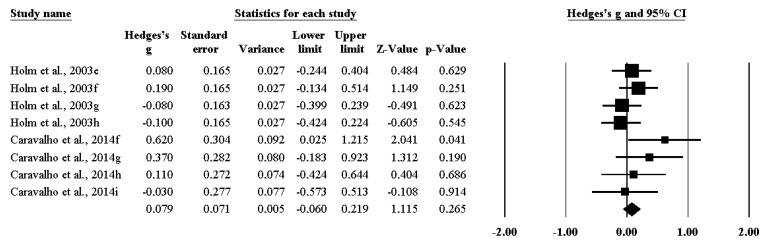
The impact of resistance training on isokinetic strength among handball players.

The impact of RT on isokinetic strength (Figure 2) was not statistically significant (ES 0.079 [95%CI −0.060–0.219], *p* = 0.265).

**Figure 3:**
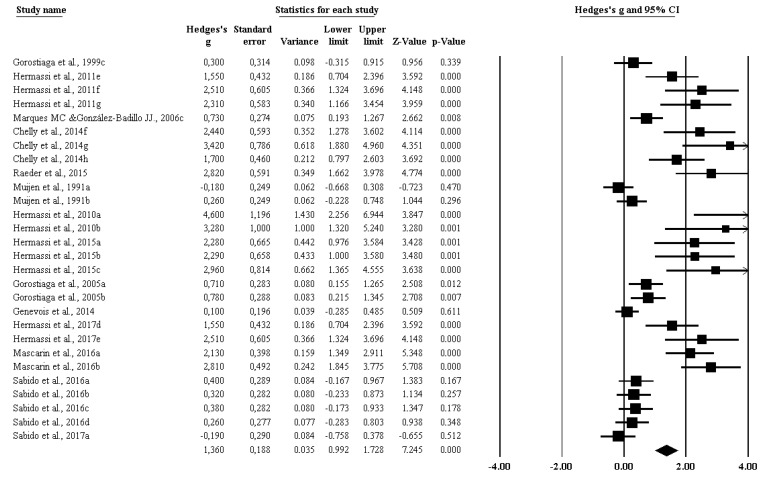
The impact of resistance training on throwing among handball players.

The impact of RT on throwing (Figure 3) (ES 1.360 [95%CI 0.992–1.728], *p* = 0.000) was significant.

**Figure 4:**
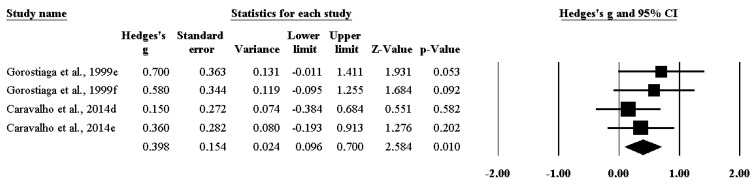
The impact of resistance training on isometric strength among handball players.

**Figure 5:**
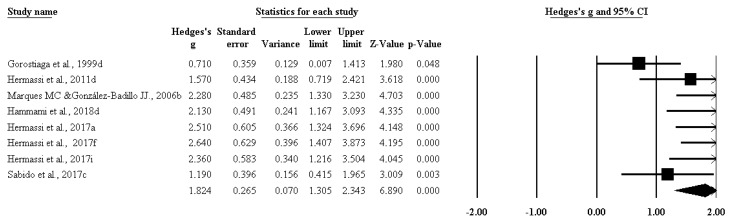
The impact of resistance training on maximal strength among handball players.

**Figure 6:**
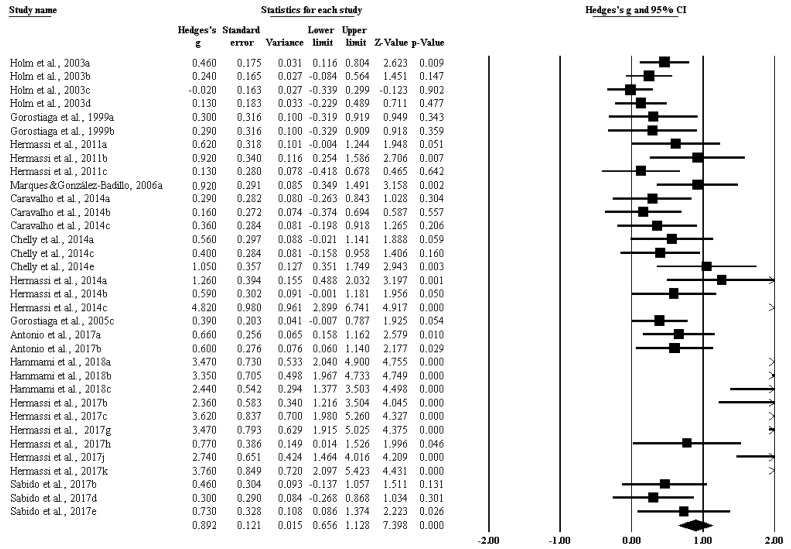
The impact of resistance training on muscle power among handball players.

Moreover, the impact of RT on isometric strength (Figure 4) (ES 0.398 [95%CI 0.096–0.700], *p* = 0.010), on maximal strength (Figure 5) (ES 1.824 [95%CI 1.305–2.343], *p* = 0.000), and on power (Figure 6) (ES 0.892 [95%CI 0.656–1.128], *p* = 0.000) was significant.

Statistically significant determinants of the impact on power were the country, gender and number of weeks, whilst determinants of the impact on throwing were publication year, country, and times of sessions per week. In all other cases, it was not possible to perform meta-regressions due to the insufficient number of available studies per covariate or due to multi-collinearity issues.

Considering the control groups, the overall ES was 0.173 ([95%CI 0.048–0.299], *p* = 0.007); 0.071 ([95%CI −0.176–0.319], *p* = 0.573) for throwing, 0.266 ([95%CI 0.144–0.388], *p* = 0.000) for power, 0.138 ([95%CI −0.188–463], *p* = 0.407) for maximal strength, and 0.163 [95%CI −0.274–0.600], *p* = 0.466) for isometric strength.

Quality assessment of the studies is reported in Supplementary Material Table S1. 

## 4. Discussion

### 4.1. Isokinetic Strength

The most significant gains can usually be verified in isometric and eccentric force production, mainly in the knee flexors [23]. These improvements can be justified by the fact that these actions are not performed with such a frequency as concentric actions, so they are more susceptible to training stimuli. Moreover, handball is a game characterized by complex movements that require constant mobility of knee extensors in order to achieve high-speed and quick changes of direction [6,7,8,26,27].

Since the knee flexors are not involved, their strength is commonly relatively low, which enables players to obtain greater strength gains after the training program. Gorostiaga et al. [15], for instance, found gains of 13% and 9% in leg extensor and flexor muscles, respectively, after a 6-week period of heavy training. Similarly, Carvalho et al. [24] showed an improvement in the peak torque of thigh muscles and concentric ratio after a 4-week heavy training program. Likewise, most strength variables increased between the two moments of assessment; however, the most significant differences were found with the knee isometric left antagonist (11.4%), eccentric left agonist (9.6%), isometric right agonist (8%) and eccentric right antagonist (6.6%).

### 4.2. Throwing Ball Velocity

Elite handball players achieve significantly higher throwing ball velocities than their lower level counterparts [28,29]—with an 8–9% advantage in elite men [15] and 10–11% advantage in elite females [26]. Hermassi et al. [7] and Gorostiaga et al. [15] noted an increase in all types of throw ball velocities (standing throw, three-step running throw and jump-throw) following 8 weeks of heavy RT. Similar improvements were obtained after 8-week bi-weekly plyometric training [5] and after an 8-week tri-weekly upper limb specific RT in handball players [25].

By increasing workloads, introducing higher throwing weights (pyramid training), accompanied with a relatively small number of repetitions and less training time, is required to obtain an optimal response. Gorostiaga et al. [15] noted a significant enhancement (*p* < 0.001) of standing throwing ball after 6 weeks of heavy upper limb RT programs. Moreover, van den Tillaar [29], comparing the benefits of various handball-training programs, found that RT was the most effective in increasing throwing ball velocity.

However, existing studies are mainly focused on concentric exercises [28,29], even though most actions required during handball imply a combination of eccentric and concentric muscular contractions, involving, for example, both concentric and eccentric phases of squat jumps [28,29].

Different studies [30] reported a significant increase by 1.2–18% in throwing ball velocity after RT (three sets of six repetitions with a load of around 85% of 1RM), with the training groups performing extra training compared to the control groups.

A combination of muscle strength, handball techniques, and competitive skills training can significantly enhance maximal and specific explosive strength of the upper limb over an 8- to 10-week training program [6,7,8,12,13,17,28]. This increase should confer players an advantage in sustaining the muscle contractions required during ball throwing, hitting, blocking, pushing, and holding [6,7,15,17].

In conclusion, throwing ball velocity can be increased positively after RT programs that incorporate, as a minimum, training two to three times per week for 5 weeks of general RT for the upper body or specific resistance protocols.

### 4.3. Isometric Strength

Gorostiaga et al. [17] showed that during the 6-week training period, the mean 1RM performance in the training group improved in the leg extensor muscles (leg press) from 126.7 kg to 142.2 kg (*p* < 0.01), and in the upper extremity muscles (pec–deck) from 36.1 kg to 44.4 kg (*p* < 0.01). This increase occurred mainly during the first 2 weeks, whereas there was only a slight improvement during the following 4 weeks of training. The addition of a 6-week period of heavy training resulted in considerable gains in the maximal muscle strength of the leg extensors (13%) and the arm muscles (23%), with minor changes in the maximal isometric force of the leg flexor muscles (9%).

A difference in strength gains between upper and lower extremity muscles can be explained by the difference in initial conditioning between knee extensor and upper body muscles, depending also on the pattern of quantity and/or intensity of daily physical use.

Interestingly, studies investigating the effects of concurrent strength and endurance training found an interference in the optimal development of muscle strength [29]. Handball training sessions and competitive games place energy production demands on the leg muscles mainly through aerobic processes.

### 4.4. Maximal Strength

Competitive performance in handball depends not only on muscle strength, but also on the ability to exert force at the speed required by this discipline. The longer contraction durations appear to be best suited for maximizing muscle strength [29]. The training programs developed for strength/power for handball players have traditionally focused on resistance exercises (i.e., squat, pullover and bench press) that primarily emphasize maximal force production [17,29].

These exercises, despite an initial explosive contraction, are performed at a slow velocity of movement and have the greatest effect on strength improvement at the slow velocity/high force segment of the force–velocity–power curve [12,13]. However, this mode of training does not appear to maximize power performance, especially in the experienced RT players.

In this context, Gorostiaga et al. [17] found that RT can improve the strength of both the upper extremity muscles (23%; *p* < 0.01) and the leg extensors (12.2%; *p* < 0.01), although no gains can be anticipated from low resistance forms of activity such as normal handball practice. Furthermore, Hermassi et al. [30] and Chelly et al. [5] reported gains for the upper and lower limbs (21.8 and 14.5%, for bench press and half-squat, respectively) even larger than those observed by Gorostiaga et al. [17] and Hermassi et al. [7], possibly due to differences in either the initial training status of the players and/or the training exercises performed.

The results of Hermassi et al. [6,7,8] are in agreement with other investigations [28] of plyometric training for the upper and lower limbs. The increased workload positively transferred to throws with a regular handball ball. According to Hermassi et al. [30], a combination of strength, handball technique, and competitive skills training significantly enhanced the maximal and specific explosive strength of the upper extremities over the 10-week program. The increase in maximal upper limb and lower limb maximal strength should give players an advantage in sustaining the forceful muscle contractions required during such actions [17].

On the other hand, Gorostiaga et al. [15] studied the effect of an entire season of play (45 weeks) on the strength–load relationships for the arm extensor muscles of elite male handball players. Performance was assessed on four occasions: the beginning (T1) of the first preparatory period, at the beginning (T2) and the end (T3) of the first competitive period, and at the end of the second competitive period (T4). Training was periodized from a high-volume, low-intensity phase during the preparatory period to a low-volume, high-intensity phase toward the competitive period. Values of 1RM bench press obtained at T3 increased significantly (*p* < 0.01) compared with T1. The greater number of weekly RT sessions could explain the higher increase in 1RM upper limb strength in this study.

Marques et al. [12] examined the effects of 12 weeks of RT (two to three sessions per week) in high-level handball players with loads ranging from 70–85% of concentric 1RM bench press. They noted a 28% increase in 1RM bench press; however, in a similar study by Hermassi et al. [30] the training group improved their 1RM bench press by only 16%. Whether assessed by 1RM pullover or bench press, moderate strength training showed gains of 24% and 6% for 1RM pullover and 1RM bench press, respectively [30]. However, such gains did not statistically surpass the gains seen in those following the control regimen, and they were significantly less than those in a similar study by other researchers [28].

### 4.5. Muscle Power

Few investigations in team handball examined the effects of RT using dynamic exercises on the peak muscle power (W_peak_) determined by means of a cycling force–velocity test [6,7]. For instance, Hermassi et al. [6] compared gains of muscle peak power, using successive eccentric–concentric exercises for the upper and lower body. Gains of absolute power for both the lower (18%; *p* < 0.01) and upper (13%; *p* < 0.01) extremities [7] were reported, in absence of significant changes in relative power to body mass for the upper limbs.

The prescribed loads during RT enhance muscle power and thus sport performance [5,29], as also shown by Hermassietal. [6], initiating feedback reflexes from the Golgi tendon organs and/or improving the synchronization of the firing motor unit [15].

Moreover, Hermassi et al. [6] investigated elite handball players participating in a 12-week in-season strength training program, with a frequency of two sessions per week (two exercises for the upper limbs, like pullover and bench press, and for the lower limbs, such as half squat). Loads were 80–95% of the personal 1RM, based on a succession of eccentric-concentric muscle contractions at a slow velocity, interspersed by rest intervals of 3–4 min between repetitions. Heavy training led to improvements in both absolute (W) (12.39%; *p* < 0.01) and relative (W/kg) (12.9%; *p* < 0.01) muscle power for the upper limbs, suggesting that muscle power gain could be attributable to an increase in the regional muscle volume of the upper limbs [6].

In fact, the average percentage increase in muscle power per unit of muscle volume (W/l) for the heavy training group (8.5%) tended to be higher than for controls (3.7%). Heavy RT may increase muscle bulk appreciably, potentially due to neuronal adaptation [29].

In fact, 12 weeks of heavy RT led to a considerable gain in muscle power (W) (12.41%; *p* < 0.001) and in the relative power (W/kg) (13.1%; *p* < 0.01) of the upper limbs, but no changes when power was expressed per liter of upper limb muscle volume (W/l) [6]. The average percentage increase in muscle power per unit of muscle volume (W/l) for the heavy training group (5.1 ± 6%) was higher than for the control group (0.5 ± 9.7%). The average percentage increase in muscle power per unit of muscle volume (W/l of the thigh) for the heavy training group (3.5 ± 4.4%) was higher than for the control group (0.1 ± 3.2%).

Although the load prescription based upon the maximizing of mechanical power output appears an attractive strategy to enhance the muscle power of the limbs, performance may be critically dependent on the ability to exert force at speeds specific to a given athletic discipline. Longer contraction durations have been associated with heavier loads, so the prescription of such loads would seem best suited to maximizing strength.

### 4.6. Strengths and Limitations

To the best of our knowledge, this is the first systematic review and meta-analysis that comprehensively and quantitatively assesses the impact of RT among handball players. However, despite its novelty, the present investigation suffers from a number of shortcomings which should be properly recognized and addressed. The main drawbacks are represented by the significant amount of heterogeneity and the evidence of publication bias that is present in many fields of the research domain.

## 5. Conclusions

Our systematic review and meta-analysis showed that RT has a significant impact in handball players in terms of maximal strength (isokinetic and isometric strength), muscle power of both upper and lower limbs, and throwing velocity. The present review has important practical implications, in that handball coaches could design conditioning protocols based on our results. However, due to the above-mentioned shortcomings, including evidence of bias and high, statistically significant heterogeneity among studies, further high-quality investigations in the field are urgently needed.

## Figures and Tables

**Table 1:** Search strategy adopted in the present systematic review and meta-analysis.

Search Strategy Item	Details
Searched keywords	handball AND “resistance training” AND (maximal strength OR force OR power OR throwing velocity OR isometric strength OR isokinetic strength)
Searched databases	PubMed/MEDLINE, the Cochrane Central Register of Controlled Trials (CENTRAL), Scopus and ISI/Web of Science
Inclusion criteria	P (Participants/population): handball athletes (either amateur or professional players), men or women, youths or adults; E (Exposures): handball players exposed to RT; C (Comparator(s)/control): other kinds of training versus RT; O (Outcomes): the effect of the RT on maximal strength, isometric or isokinetic strength, power, throwing velocity; S (Study design): Any experimental study design.
Exclusion criteria	P (Participants/population): sports athletes other than handball players; E (Exposures): handball players exposed to training other than RT or combined with RT, in which it was not possible to pull out the single effects of RT; C (Comparator(s)/control): other kinds of training rather than RT versus RT; O (Outcomes): the effect of the RT on other variables; S (Study design): expert opinion; comment/commentary; editorial/letter to editor; review.

Abbreviation: resistance training (RT).

**Table 2:** Main characteristics of included studies.

Authors	Year	Country	Randomized or Not (R, NR)	Sample	Drop-Out Rate	Age	Gender (in Percentage)	Anthropometric Features of Sample (Height, Body Mass, BMI, etc.)	Sport	Level (International, National, Elite)	Type of Training	Duration of Training	Variable Measured	Statistical Test Used
Gorostiaga et al. [17]	2005	Spain	Randomized	15	*0*	31 ± 4	100% men	Body mass: 95.6 ± 14.3; Body fat (%): 14.9 ± 4.2; Height (cm): 188 ± 7	Handball	Elite	Maximal strength of the upper extremity was assessed using one repetition concentric maximal bench press action	45-weeks in season	Vertical jumping height, throwing velocity, muscle power output; maximal strength of upper and lower limb	ANOVA with repeated measures was used to determine the differences between tests. When a significant F value was achieved, appropriate Scheffe’s post-hoc tests procedures were used to locate the difference between means
Holm et al. [23]	2004	Norway	Randomized	35	*0*	23 ± 2.5	100% women	Body mass was 69.2 ±7.3 kg	Handball	Elite division	Neuromuscular training	Three times a week over 8 weeks	Balance, muscle strength of lower limb	A 3 (time) × 2 (leg) analysis of variance for repeated measures (with the least significant difference post hoc test) was used to calculate differences from one test to the next over the study period
Oxyzoglou et al. [22]	2007	Greece	Randomized	51	*0*	13.7 ± 1.5	100% men	Height = 168.06 ±7.8 cm; weight = 56.90 ± 10.35 kg	Handball	Pre-adolescent athletes	Various shooting throws with horizontal and vertical jumps of different height	Three sessions/week 60 min 6-month	Long jump, vertical jump, throwing of medicine ball, strength of right hand grip, strength of left hand grip, hanging from a horizontal bar, body sit- up	An analysis of covariance (ANCOVA) was performed to examine the differences between groups in post-training values where the pre-training mean was used as a covariate
Ettema et al. [21]	2008	Norway	Randomized	19	6 players	18.1 ± 2.1	100% women	Body mass 64.0 ± 7 kg, height 1.67 ± 0.03 m	Handball	Sub-elite	Pulley device mimicking throwing kinematics at 85% of 1RM	Three sessions per week over 8 weeks	Throwing velocity	A two-way ANOVA for repeated measures was used
Gorostiaga et al. [15]	1999	Spain	Not mentioned	24	NO	15.1 ± 0.7	100% men	Body mass 62.4 ± 7 kg, height 1.73 ± 0.05 m, and body fat 11.3 ± 3.1%)	Handball	Sub-elite	Heavy-resistance weight lifting 40% to 90% RM: (bench press, half squat, knee fexion curl, leg press and pec-deck)	Two sessions per week over 6 weeks	Throwing ball velocity, squat jump, countermovement jump, 1RM leg press, 1RM pec-deck	One-way ANOVA
Hermassi et al. [6]	2010	Tunisia	Randomized	26	NO	20 ± 0.6	100% men	Body mass 85.0 ±13.2 kg, height 1.86 ± 0.06 m, and body fat 13.7 ± 2.4%)	Handball	Elite	Heavy resistance training group (80% to 95% of 1RM) and moderate resistance training group (55% to 75% of 1RM)	Two sessions per week over 10 weeks	Upper limb power, handball throwing velocity, 1RM bench press, 1RM pullover	A two-way analysis of variance (ANOVA) with repeated measure (group X time)
Hermassi et al. [7]	2011	Tunisia	Randomized	24	0	24 ± 0.7	100% men	Height 1.83 ± 0.08 m, body mass 81 ± 12 kg, body fat 13.2 ± 1.3%	Handball	Elite	Heavy resistance training (80% to 95% of 1RM) group vs. control group	Two sessions per week over 8 weeks	Peak power (cycle ergometer), squat jump, countermovement jump, sprint tests, 1RM bench press, 1RM pullover, 1RM back half squat test	Training effects were assessed by a one-way analysis of variance with repeated measure (group X time).
Hermassi et al. [8]	2014	Tunisia	Randomized	24	0	20 ± 0.3	100% men	Body mass: 89.1 ± 2.1 kg, height: 1.88 ± 0.07 m, body fat: 13.2 ± 1.3%)	Handball	Elite	Plyometric training	Two sessions per week over 8 weeks	Leg power (cycle ergometer), squat jump, countermovement jump	To compare the effects of the plyometric training, a mixed design 2 (test occasion: pre–post: repeated measures) × 2 (group: plyometric and control) Analysis of variance (ANOVA) for each performance test variable was used. In addition, a one-way ANOVA with repeated measures was conducted for each group to show if changes from pre- to post-test in each group were significant.
Hermassi et al. [25]	2015	Tunisia	Randomized	34	0	18 ± 0.5	100% men	Body mass: 80.6 ± 5.5 kg, height: 1.80 ± 5.1 cm, body fat: 13.4 ± 0.6%)	Handball	Elite	Group 1: throwing with medicine ball of 3 kg, Group 2: throwing with regular ball, control group	Three sessions per week over 8 weeks	1RM bench press, 1RM pull over+ ball throwing velocity and ball throwing distance	To compare the effects of the training protocols, a mixed-design 2 (test occasion: pre–post: repeated measures) 3 (group: control, regular throwing, and resistance training) ANOVA on each variable was used
Marques & González-Badillo [14]	2006	Spain	Not mentioned	16	0	18 to 29 years old (23.1 ± 4.7)	100% men	Body mass: 84.8 ± 13.1 kg, height: 1.84.2 ±13.1 cm	Handball	High level (international and national)	Strength and power training: bench press and parallel squat (70% to 95% 1RM), CMJ and sprint training	Two to three sessions per week over 12 weeks	1RM bench press, 4RM parallel squat, CMJ, sprint tests (30m), ball throwing velocity	A repeated-measures analysis of variance with Bonferroni adjustment was used to assess gains or losses
Ignjatovic et al. [13]	2012	Serbia	Randomized	21	0	16.9 ± 1.2	100% female	Not mentioned	Handball	Elite	Medicine ball training	Two sessions per week over 12 weeks	Muscle strength: 1RM bench press, 1RM shoulder press, for muscle power: 30% RM bench press, 50% RM bench press, 30% RM shoulder press, 50% RM shoulder press, ball throwing distance	Changes in muscle power were analyzed separately using 2 × 2 (treatment × time) repeated measure analysis of variance (ANOVA)
Carvalho et al. [24]	2014	Portugal	Not mentioned	12	0	21.6 ± 1.73	100% men	Body height 183.9 ± 0.09 cm; body mass 81.7 ± 8.3 kg)	Handball	Semi-professional	Strength, plyometric training	Three sessions per week over 12 weeks	Maximum dynamic and isometric strength, squat jump, countermovement jump, 40 consecutive jumps	A repeated measurement paired-samples *t*-test was used to assess the training effects within groups
Chelly et al. [5]	2014	Tunisia	Randomized	23	0	17.4 ± 0.5	100% men	Body mass: 79.9 ± 11.5 kg, height: 1.79 ± 6.19 m, body fat: 13.8 ± 2.1%	Handball	Elite	Plyometric training on upper and lower limb	Two sessions per week over 8 weeks	Force–velocity test for upper limbs (cycle ergometer), force–velocity test for lower limbs (cycle ergometer), squat jump, countermovement jump, throwing ball test	Training-related effects were assessed by two-way analyses of variance with repeated measures (grouped three times)
Raeder et al. [9]	2015	Spain	Randomized	28	0	20.8 ± 3.3	100% women	Height: 170.5 ± 5.6 cm, body mass: 65.2 ± 8.0 kg	Handball	Amateur	Medicine ball throws	Three sessions per week over 6 weeks	Throwing ball velocity, isokinetic strength: peak torques of shoulder internal and external rotators	A two-factor analysis of variance for repeated measurements was calculated to determine differences between the measurement points (maineffect for time), between the groups (main effect for group), and for the changeover in time in response to the different training interventions (three time interactions)
Genevois et al. [10]	2014	France	Randomized	25	0	15.8 ± 0.8	100% women	Height 169.8 ± 5 cm, body mass 59.3 ± 9.2 kg	Handball	Elite	Sling exercise for shoulder external/internal rotators and scapular retraction	6 weeks	Maximal throwing velocity, strength of shoulder External/internal rotators and scapular retraction	ANOVAs with two internal factors (training condition and training period)
Granados et al. [26]	2007	Spain	Randomized	16	0	23.1 ± 4	100% women	Body mass (kg): 69.6 ± 8.4; Body fat (%) 21.1 ± 5.3; Height (cm): 175 ± 6	Handball	Elite	Dynamic parallel squat lift and bench press and power clean and pullover: the load in the squat lift exercises (three to four sets, three to four reps) ranged from 60% to 110% of the load, in parallel squat actions. This corresponds to a load ranging from approximately 36% to 77% 1RM in the squat lift exercise	40 weeks in season	Vertical jumping height, throwing velocity, muscle power output; maximal strength of upper and lower limb	One-way analysis of variance with repeated measures was used to determine the differences between tests. When a significant F value was achieved, appropriate Scheffe’s post-hoc test procedures were used to locate the difference between means
Toumi et al. [19]	2004	France	Randomized	22	0	21 ± 2	100% men	Body mass (kg): 81 ± 6; Height (cm): 181 ± 8	Handball	Sub-elite	Weight training group: leg press machine at 70% of maximal isometric force output. Combined training group: leg press machine at 70% of maximal isometric force output, jumping exercises	Three sessions per week over 6 weeks	Squat jump, countermovement jump, maximal isometric force, maximal power	Two-way ANOVA (repeated measures on one factor) was used for the statistical analysis
van Muijen et al. [20]	1991	Netherlands	Randomized	56	11	23 ± 4	100% women	Body mass (kg): 65.3 ± 5.7; Body fat (%) 27.5 ± 2.9; Height (cm): 169.5 ± 5.7	Handball	Sub-elite	Group 1: training with normal hand balls (400 g). Group 2: training with heavy balls (500 g). Group 3: training with light balls (300 g).	Two sessions per week over 8 weeks	Throwing ball velocity, maximal isokinetic torque of elbow extensors and medial shoulder rotators	Student’s *t*-test

## Supplementary Materials

The following are available online at https://www.mdpi.com/1660-4601/17/8/2663/s1, Table S1: Quality assessment of studies included in the present systematic review and meta-analysis.
