# Supplementary material for: Resistance Training and Handball Players’ Isokinetic, Isometric and Maximal Strength, Muscle Power and Throwing Ball Velocity: A Systematic Review and Meta-Analysis
Source: Int J Environ Res Public Health. 2020 Apr 13;17(8):2663. doi: 10.3390/ijerph17082663 (PMC7215414; doi:10.3390/ijerph17082663)
Supplement: Supplementary file 1 [file ijerph-17-02663-s001.pdf]

Gorostiaga et al 2005	Score
Reporting	
1. Is the hypothesis/aim/objective of the study clearly described?	1
2. Are the main outcomes to be measured clearly described in the introduction or methods section?	1
3. Are the characteristics of the patients included in the study clearly described?	1
4. Are the intervention of interest clearly described?	1
5. Are the distributions of principal confounder in each group of subjects to be compared clearly described?	1
6. Are the main findings of the study clearly described?	1
7. Does the study provide estimates of the random variability in the data for the main outcomes?	1
8. Have all of the important adverse events that may be a consequence of the intervention been reported?	0
9. Have the characteristics of patients lost to follow-up been described?	0
10. Have actual probability values been reported for the main outcomes except where the probability value is less than 0.001?	0
External validity	
11. Were the subjects asked to participate in the study representative of the entire sample from which they were recruited?	1
12. Were those subjects who were prepared to participated representative of the entire population from which they were recruited?	0
13. Were the staff, places, and facilities where the patients were treated, representative of the treatment the majority of patients receive?	1
Study bias	
14. Was an attempt made to blind study subjects to the intervention they received?	0
15. Was an attempt made to blind those measuring the main outcomes of the intervention?	0
16. If any of the results of the study were based on “data dredging”, was this made clear?	0
17. In trials and cohort studies, do the analyses adjust for different lengths of follow-up of patients, or in case-control studies, is the time period between the intervention and outcome the same for cases and controls?	1
18. Were the statistical tests used to assess the main outcomes appropriate?	1
19. Was compliance with the intervention/s reliable?	0
20. Were the main outcome measures used accurate (valid and reliable?	0
Confounding (selection bias)	
21. Were the patients in different intervention groups (trials and cohort studies) or were the cases and controls (case-control studies) recruited from the same population?	1
22. Were study subjects in different intervention groups (trials and	1

cohort studies) or were the cases and controls (case-control studies) recruited over the same time?	
23. Were study subjects randomized to intervention groups?	1
24. Was the randomized intervention assignment concealed from both patients and health care staff until recruitment was complete and irrevocable?	0
25. Was there adequate adjustment for confounding in the analyses from which the main findings were drawn?	0
26. Were losses of patients to follow-up taken into account?	0
Power	
27. Did the study have sufficient power to detect a clinically important effect where the probability value for a difference being due to chance is < 5%	1
Holm et al 2004	Score
Reporting	
1. Is the hypothesis/aim/objective of the study clearly described?	1
2. Are the main outcomes to be measured clearly described in the introduction or methods section?	1
3. Are the characteristics of the patients included in the study clearly described?	1
4. Are the intervention of interest clearly described?	1
5. Are the distributions of principal confounder in each group of subjects to be compared clearly described?	1
6. Are the main findings of the study clearly described?	1
7. Does the study provide estimates of the random variability in the data for the main outcomes?	1
8. Have all of the important adverse events that may be a consequence of the intervention been reported?	0
9. Have the characteristics of patients lost to follow-up been described?	0
10. Have actual probability values been reported for the main outcomes except where the probability value is less than 0.001?	0
External validity	
11. Were the subjects asked to participate in the study representative of the entire sample from which they were recruited?	1
12. Were those subjects who were prepared to participated representative of the entire population from which they were recruited?	0
13. Were the staff, places, and facilities where the patients were treated, representative of the treatment the majority of patients receive?	1
Study bias	
14. Was an attempt made to blind study subjects to the intervention they received?	0
15. Was an attempt made to blind those measuring the main outcomes of the intervention?	0
16. If any of the results of the study were based on “data dredging”, was this made clear?	0
17. In trials and cohort studies, do the analyses adjust for different lengths of follow-up of patients, or in case-control studies, is the	1

time period between the intervention and outcome the same for cases and controls?	
18. Were the statistical tests used to assess the main outcomes appropriate?	1
19. Was compliance with the intervention/s reliable?	0
20. Were the main outcome measures used accurate (valid and reliable?	0
Confounding (selection bias)	
21. Were the patients in different intervention groups (trials and cohort studies) or were the cases and controls (case-control studies) recruited from the same population?	1
22. Were study subjects in different intervention groups (trials and cohort studies) or were the cases and controls (case-control studies) recruited over the same time?	1
23. Were study subjects randomized to intervention groups?	1
24. Was the randomized intervention assignment concealed from both patients and health care staff until recruitment was complete and irrevocable?	0
25. Was there adequate adjustment for confounding in the analyses from which the main findings were drawn?	0
26. Were losses of patients to follow-up taken into account?	0
Power	
27. Did the study have sufficient power to detect a clinically important effect where the probability value for a difference being due to chance is < 5%	1
Oxyzoglou et al 2007	Score
Reporting	
1. Is the hypothesis/aim/objective of the study clearly described?	1
2. Are the main outcomes to be measured clearly described in the introduction or methods section?	1
3. Are the characteristics of the patients included in the study clearly described?	1
4. Are the intervention of interest clearly described?	1
5. Are the distributions of principal confounder in each group of subjects to be compared clearly described?	1
6. Are the main findings of the study clearly described?	1
7. Does the study provide estimates of the random variability in the data for the main outcomes?	1
8. Have all of the important adverse events that may be a consequence of the intervention been reported?	0
9. Have the characteristics of patients lost to follow-up been described?	1
10. Have actual probability values been reported for the main outcomes except where the probability value is less than 0.001?	0
External validity	
11. Were the subjects asked to participate in the study representative of the entire sample from which they were recruited?	1
12. Were those subjects who were prepared to participated representative of the entire population from which they were recruited?	0

13. Were the staff, places, and facilities where the patients were treated, representative of the treatment the majority of patients receive?	1
Study bias	
14. Was an attempt made to blind study subjects to the intervention they received?	0
15. Was an attempt made to blind those measuring the main outcomes of the intervention?	0
16. If any of the results of the study were based on "data dredging", was this made clear?	0
17. In trials and cohort studies, do the analyses adjust for different lengths of follow-up of patients, or in case-control studies, is the time period between the intervention and outcome the same for cases and controls?	1
18. Were the statistical tests used to assess the main outcomes appropriate?	1
19. Was compliance with the intervention/s reliable?	0
20. Were the main outcome measures used accurate (valid and reliable?	0
Confounding (selection bias)	
21. Were the patients in different intervention groups (trials and cohort studies) or were the cases and controls (case-control studies) recruited from the same population?	1
22. Were study subjects in different intervention groups (trials and cohort studies) or were the cases and controls (case-control studies) recruited over the same time?	1
23. Were study subjects randomized to intervention groups?	1
24. Was the randomized intervention assignment concealed from both patients and health care staff until recruitment was complete and irrevocable?	0
25. Was there adequate adjustment for confounding in the analyses from which the main findings were drawn?	0
26. Were losses of patients to follow-up taken into account?	0
Power	
27. Did the study have sufficient power to detect a clinically important effect where the probability value for a difference being due to chance is < 5%	1
Ettema et al 2008	Score
Reporting	
1. Is the hypothesis/aim/objective of the study clearly described?	1
2. Are the main outcomes to be measured clearly described in the introduction or methods section?	1
3. Are the characteristics of the patients included in the study clearly described?	1
4. Are the intervention of interest clearly described?	1
5. Are the distributions of principal confounder in each group of subjects to be compared clearly described?	1
6. Are the main findings of the study clearly described?	1
7. Does the study provide estimates of the random variability in the data for the main outcomes?	1

8. Have all of the important adverse events that may be a consequence of the intervention been reported?	0
9. Have the characteristics of patients lost to follow-up been described?	1
10. Have actual probability values been reported for the main outcomes except where the probability value is less than 0.001?	0
External validity	
11. Were the subjects asked to participate in the study representative of the entire sample from which they were recruited?	0
12. Were those subjects who were prepared to participated representative of the entire population from which they were recruited?	0
13. Were the staff, places, and facilities where the patients were treated, representative of the treatment the majority of patients receive?	1
Study bias	
14. Was an attempt made to blind study subjects to the intervention they received?	0
15. Was an attempt made to blind those measuring the main outcomes of the intervention?	0
16. If any of the results of the study were based on "data dredging", was this made clear?	0
17. In trials and cohort studies, do the analyses adjust for different lengths of follow-up of patients, or in case-control studies, is the time period between the intervention and outcome the same for cases and controls?	1
18. Were the statistical tests used to assess the main outcomes appropriate?	1
19. Was compliance with the intervention/s reliable?	0
20. Were the main outcome measures used accurate (valid and reliable?	0
Confounding (selection bias)	
21. Were the patients in different intervention groups (trials and cohort studies) or were the cases and controls (case-control studies) recruited from the same population?	1
22. Were study subjects in different intervention groups (trials and cohort studies) or were the cases and controls (case-control studies) recruited over the same time?	1
23. Were study subjects randomized to intervention groups?	1
24. Was the randomized intervention assignment concealed from both patients and health care staff until recruitment was complete and irrevocable?	0
25. Was there adequate adjustment for confounding in the analyses from which the main findings were drawn?	0
26. Were losses of patients to follow-up taken into account?	0
Power	
27. Did the study have sufficient power to detect a clinically important effect where the probability value for a difference being due to chance is < 5%	1
Gorostiaga et al 1999	Score

Reporting	
1. Is the hypothesis/aim/objective of the study clearly described?	1
2. Are the main outcomes to be measured clearly described in the introduction or methods section?	1
3. Are the characteristics of the patients included in the study clearly described?	1
4. Are the intervention of interest clearly described?	1
5. Are the distributions of principal confounder in each group of subjects to be compared clearly described?	1
6. Are the main findings of the study clearly described?	1
7. Does the study provide estimates of the random variability in the data for the main outcomes?	1
8. Have all of the important adverse events that may be a consequence of the intervention been reported?	0
9. Have the characteristics of patients lost to follow-up been described?	1
10. Have actual probability values been reported for the main outcomes except where the probability value is less than 0.001?	1
External validity	
11. Were the subjects asked to participate in the study representative of the entire sample from which they were recruited?	1
12. Were those subjects who were prepared to participated representative of the entire population from which they were recruited?	0
13. Were the staff, places, and facilities where the patients were treated, representative of the treatment the majority of patients receive?	1
Study bias	
14. Was an attempt made to blind study subjects to the intervention they received?	0
15. Was an attempt made to blind those measuring the main outcomes of the intervention?	0
16. If any of the results of the study were based on “data dredging”, was this made clear?	0
17. In trials and cohort studies, do the analyses adjust for different lengths of follow-up of patients, or in case-control studies, is the time period between the intervention and outcome the same for cases and controls?	1
18. Were the statistical tests used to assess the main outcomes appropriate?	1
19. Was compliance with the intervention/s reliable?	0
20. Were the main outcome measures used accurate (valid and reliable?	0
Confounding (selection bias)	
21. Were the patients in different intervention groups (trials and cohort studies) or were the cases and controls (case-control studies) recruited from the same population?	1
22. Were study subjects in different intervention groups (trials and cohort studies) or were the cases and controls (case-control studies) recruited over the same time?	1

23. Were study subjects randomized to intervention groups?	0
24. Was the randomized intervention assignment concealed from both patients and health care staff until recruitment was complete and irrevocable?	0
25. Was there adequate adjustment for confounding in the analyses from which the main findings were drawn?	0
26. Were losses of patients to follow-up taken into account?	0
Power	
27. Did the study have sufficient power to detect a clinically important effect where the probability value for a difference being due to chance is < 5%	1
Hermassi et al 2010	Score
Reporting	
1. Is the hypothesis/aim/objective of the study clearly described?	1
2. Are the main outcomes to be measured clearly described in the introduction or methods section?	1
3. Are the characteristics of the patients included in the study clearly described?	1
4. Are the intervention of interest clearly described?	1
5. Are the distributions of principal confounder in each group of subjects to be compared clearly described?	1
6. Are the main findings of the study clearly described?	1
7. Does the study provide estimates of the random variability in the data for the main outcomes?	1
8. Have all of the important adverse events that may be a consequence of the intervention been reported?	0
9. Have the characteristics of patients lost to follow-up been described?	0
10. Have actual probability values been reported for the main outcomes except where the probability value is less than 0.001?	1
External validity	
11. Were the subjects asked to participate in the study representative of the entire sample from which they were recruited?	1
12. Were those subjects who were prepared to participated representative of the entire population from which they were recruited?	0
13. Were the staff, places, and facilities where the patients were treated, representative of the treatment the majority of patients receive?	1
Study bias	
14. Was an attempt made to blind study subjects to the intervention they received?	0
15. Was an attempt made to blind those measuring the main outcomes of the intervention?	0
16. If any of the results of the study were based on "data dredging", was this made clear?	0
17. In trials and cohort studies, do the analyses adjust for different lengths of follow-up of patients, or in case-control studies, is the time period between the intervention and outcome the same for cases and controls?	1

18. Were the statistical tests used to assess the main outcomes appropriate?	1
19. Was compliance with the intervention/s reliable?	0
20. Were the main outcome measures used accurate (valid and reliable?	1
Confounding (selection bias)	
21. Were the patients in different intervention groups (trials and cohort studies) or were the cases and controls (case-control studies) recruited from the same population?	1
22. Were study subjects in different intervention groups (trials and cohort studies) or were the cases and controls (case-control studies) recruited over the same time?	1
23. Were study subjects randomized to intervention groups?	1
24. Was the randomized intervention assignment concealed from both patients and health care staff until recruitment was complete and irrevocable?	0
25. Was there adequate adjustment for confounding in the analyses from which the main findings were drawn?	0
26. Were losses of patients to follow-up taken into account?	0
Power	
27. Did the study have sufficient power to detect a clinically important effect where the probability value for a difference being due to chance is < 5%	1
Hermassi et al 2011	Score
Reporting	
1. Is the hypothesis/aim/objective of the study clearly described?	1
2. Are the main outcomes to be measured clearly described in the introduction or methods section?	1
3. Are the characteristics of the patients included in the study clearly described?	1
4. Are the intervention of interest clearly described?	1
5. Are the distributions of principal confounder in each group of subjects to be compared clearly described?	1
6. Are the main findings of the study clearly described?	1
7. Does the study provide estimates of the random variability in the data for the main outcomes?	1
8. Have all of the important adverse events that may be a consequence of the intervention been reported?	0
9. Have the characteristics of patients lost to follow-up been described?	0
10. Have actual probability values been reported for the main outcomes except where the probability value is less than 0.001?	1
External validity	
11. Were the subjects asked to participate in the study representative of the entire sample from which they were recruited?	1
12. Were those subjects who were prepared to participated representative of the entire population from which they were recruited?	0
13. Were the staff, places, and facilities where the patients were treated, representative of the treatment the majority of patients	1

receive?	
Study bias	
14. Was an attempt made to blind study subjects to the intervention they received?	0
15. Was an attempt made to blind those measuring the main outcomes of the intervention?	0
16. If any of the results of the study were based on "data dredging", was this made clear?	0
17. In trials and cohort studies, do the analyses adjust for different lengths of follow-up of patients, or in case-control studies, is the time period between the intervention and outcome the same for cases and controls?	1
18. Were the statistical tests used to assess the main outcomes appropriate?	1
19. Was compliance with the intervention/s reliable?	0
20. Were the main outcome measures used accurate (valid and reliable?	1
Confounding (selection bias)	
21. Were the patients in different intervention groups (trials and cohort studies) or were the cases and controls (case-control studies) recruited from the same population?	1
22. Were study subjects in different intervention groups (trials and cohort studies) or were the cases and controls (case-control studies) recruited over the same time?	1
23. Were study subjects randomized to intervention groups?	1
24. Was the randomized intervention assignment concealed from both patients and health care staff until recruitment was complete and irrevocable?	0
25. Was there adequate adjustment for confounding in the analyses from which the main findings were drawn?	0
26. Were losses of patients to follow-up taken into account?	0
Power	
27. Did the study have sufficient power to detect a clinically important effect where the probability value for a difference being due to chance is < 5%	1
Hermassi et al 2014	Score
Reporting	
1. Is the hypothesis/aim/objective of the study clearly described?	1
2. Are the main outcomes to be measured clearly described in the introduction or methods section?	1
3. Are the characteristics of the patients included in the study clearly described?	1
4. Are the intervention of interest clearly described?	1
5. Are the distributions of principal confounder in each group of subjects to be compared clearly described?	1
6. Are the main findings of the study clearly described?	1
7. Does the study provide estimates of the random variability in the data for the main outcomes?	1
8. Have all of the important adverse events that may be a consequence of the intervention been reported?	0

9. Have the characteristics of patients lost to follow-up been described?	0
10. Have actual probability values been reported for the main outcomes except where the probability value is less than 0.001?	0
External validity	
11. Were the subjects asked to participate in the study representative of the entire sample from which they were recruited?	1
12. Were those subjects who were prepared to participated representative of the entire population from which they were recruited?	0
13. Were the staff, places, and facilities where the patients were treated, representative of the treatment the majority of patients receive?	1
Study bias	
14. Was an attempt made to blind study subjects to the intervention they received?	0
15. Was an attempt made to blind those measuring the main outcomes of the intervention?	0
16. If any of the results of the study were based on "data dredging", was this made clear?	0
17. In trials and cohort studies, do the analyses adjust for different lengths of follow-up of patients, or in case-control studies, is the time period between the intervention and outcome the same for cases and controls?	1
18. Were the statistical tests used to assess the main outcomes appropriate?	1
19. Was compliance with the intervention/s reliable?	0
20. Were the main outcome measures used accurate (valid and reliable?	0
Confounding (selection bias)	
21. Were the patients in different intervention groups (trials and cohort studies) or were the cases and controls (case-control studies) recruited from the same population?	1
22. Were study subjects in different intervention groups (trials and cohort studies) or were the cases and controls (case-control studies) recruited over the same time?	1
23. Were study subjects randomized to intervention groups?	1
24. Was the randomized intervention assignment concealed from both patients and health care staff until recruitment was complete and irrevocable?	0
25. Was there adequate adjustment for confounding in the analyses from which the main findings were drawn?	0
26. Were losses of patients to follow-up taken into account?	0
Power	
27. Did the study have sufficient power to detect a clinically important effect where the probability value for a difference being due to chance is < 5%	1
Hermassi et al 2015	Score
Reporting	
1. Is the hypothesis/aim/objective of the study clearly described?	1

2. Are the main outcomes to be measured clearly described in the introduction or methods section?	1
3. Are the characteristics of the patients included in the study clearly described?	1
4. Are the intervention of interest clearly described?	1
5. Are the distributions of principal confounder in each group of subjects to be compared clearly described?	1
6. Are the main findings of the study clearly described?	1
7. Does the study provide estimates of the random variability in the data for the main outcomes?	1
8. Have all of the important adverse events that may be a consequence of the intervention been reported?	0
9. Have the characteristics of patients lost to follow-up been described?	0
10. Have actual probability values been reported for the main outcomes except where the probability value is less than 0.001?	1
External validity	
11. Were the subjects asked to participate in the study representative of the entire sample from which they were recruited?	1
12. Were those subjects who were prepared to participated representative of the entire population from which they were recruited?	0
13. Were the staff, places, and facilities where the patients were treated, representative of the treatment the majority of patients receive?	1
Study bias	
14. Was an attempt made to blind study subjects to the intervention they received?	0
15. Was an attempt made to blind those measuring the main outcomes of the intervention?	0
16. If any of the results of the study were based on "data dredging", was this made clear?	0
17. In trials and cohort studies, do the analyses adjust for different lengths of follow-up of patients, or in case-control studies, is the time period between the intervention and outcome the same for cases and controls?	1
18. Were the statistical tests used to assess the main outcomes appropriate?	1
19. Was compliance with the intervention/s reliable?	0
20. Were the main outcome measures used accurate (valid and reliable?	1
Confounding (selection bias)	
21. Were the patients in different intervention groups (trials and cohort studies) or were the cases and controls (case-control studies) recruited from the same population?	1
22. Were study subjects in different intervention groups (trials and cohort studies) or were the cases and controls (case-control studies) recruited over the same time?	1
23. Were study subjects randomized to intervention groups?	1
24. Was the randomized intervention assignment concealed from both	0

patients and health care staff until recruitment was complete and irrevocable?	
25. Was there adequate adjustment for confounding in the analyses from which the main findings were drawn?	0
26. Were losses of patients to follow-up taken into account?	0
Power	
27. Did the study have sufficient power to detect a clinically important effect where the probability value for a difference being due to chance is < 5%	1
Marques &González-Badillo 2006	Score
Reporting	
1. Is the hypothesis/aim/objective of the study clearly described?	1
2. Are the main outcomes to be measured clearly described in the introduction or methods section?	1
3. Are the characteristics of the patients included in the study clearly described?	1
4. Are the intervention of interest clearly described?	1
5. Are the distributions of principal confounder in each group of subjects to be compared clearly described?	1
6. Are the main findings of the study clearly described?	1
7. Does the study provide estimates of the random variability in the data for the main outcomes?	1
8. Have all of the important adverse events that may be a consequence of the intervention been reported?	0
9. Have the characteristics of patients lost to follow-up been described?	1
10. Have actual probability values been reported for the main outcomes except where the probability value is less than 0.001?	0
External validity	
11. Were the subjects asked to participate in the study representative of the entire sample from which they were recruited?	1
12. Were those subjects who were prepared to participated representative of the entire population from which they were recruited?	0
13. Were the staff, places, and facilities where the patients were treated, representative of the treatment the majority of patients receive?	1
Study bias	
14. Was an attempt made to blind study subjects to the intervention they received?	0
15. Was an attempt made to blind those measuring the main outcomes of the intervention?	0
16. If any of the results of the study were based on “data dredging”, was this made clear?	0
17. In trials and cohort studies, do the analyses adjust for different lengths of follow-up of patients, or in case-control studies, is the time period between the intervention and outcome the same for cases and controls?	1
18. Were the statistical tests used to assess the main outcomes appropriate?	1

19. Was compliance with the intervention/s reliable?	0
20. Were the main outcome measures used accurate (valid and reliable?	0
Confounding (selection bias)	
21. Were the patients in different intervention groups (trials and cohort studies) or were the cases and controls (case-control studies) recruited from the same population?	1
22. Were study subjects in different intervention groups (trials and cohort studies) or were the cases and controls (case-control studies) recruited over the same time?	1
23. Were study subjects randomized to intervention groups?	1
24. Was the randomized intervention assignment concealed from both patients and health care staff until recruitment was complete and irrevocable?	0
25. Was there adequate adjustment for confounding in the analyses from which the main findings were drawn?	0
26. Were losses of patients to follow-up taken into account?	0
Power	
27. Did the study have sufficient power to detect a clinically important effect where the probability value for a difference being due to chance is < 5%	1
Ignjatovic et al 2012	Score
Reporting	
1. Is the hypothesis/aim/objective of the study clearly described?	1
2. Are the main outcomes to be measured clearly described in the introduction or methods section?	1
3. Are the characteristics of the patients included in the study clearly described?	1
4. Are the intervention of interest clearly described?	1
5. Are the distributions of principal confounder in each group of subjects to be compared clearly described?	1
6. Are the main findings of the study clearly described?	1
7. Does the study provide estimates of the random variability in the data for the main outcomes?	1
8. Have all of the important adverse events that may be a consequence of the intervention been reported?	0
9. Have the characteristics of patients lost to follow-up been described?	1
10. Have actual probability values been reported for the main outcomes except where the probability value is less than 0.001?	0
External validity	
11. Were the subjects asked to participate in the study representative of the entire sample from which they were recruited?	1
12. Were those subjects who were prepared to participated representative of the entire population from which they were recruited?	0
13. Were the staff, places, and facilities where the patients were treated, representative of the treatment the majority of patients receive?	1
Study bias	

14. Was an attempt made to blind study subjects to the intervention they received?	0
15. Was an attempt made to blind those measuring the main outcomes of the intervention?	0
16. If any of the results of the study were based on “data dredging”, was this made clear?	0
17. In trials and cohort studies, do the analyses adjust for different lengths of follow-up of patients, or in case-control studies, is the time period between the intervention and outcome the same for cases and controls?	1
18. Were the statistical tests used to assess the main outcomes appropriate?	1
19. Was compliance with the intervention/s reliable?	0
20. Were the main outcome measures used accurate (valid and reliable?	1
Confounding (selection bias)	
21. Were the patients in different intervention groups (trials and cohort studies) or were the cases and controls (case-control studies) recruited from the same population?	1
22. Were study subjects in different intervention groups (trials and cohort studies) or were the cases and controls (case-control studies) recruited over the same time?	1
23. Were study subjects randomized to intervention groups?	1
24. Was the randomized intervention assignment concealed from both patients and health care staff until recruitment was complete and irrevocable?	0
25. Was there adequate adjustment for confounding in the analyses from which the main findings were drawn?	0
26. Were losses of patients to follow-up taken into account?	0
Power	
27. Did the study have sufficient power to detect a clinically important effect where the probability value for a difference being due to chance is < 5%	1
Caravalho et al 2014	Score
Reporting	
1. Is the hypothesis/aim/objective of the study clearly described?	1
2. Are the main outcomes to be measured clearly described in the introduction or methods section?	1
3. Are the characteristics of the patients included in the study clearly described?	1
4. Are the intervention of interest clearly described?	1
5. Are the distributions of principal confounder in each group of subjects to be compared clearly described?	1
6. Are the main findings of the study clearly described?	1
7. Does the study provide estimates of the random variability in the data for the main outcomes?	1
8. Have all of the important adverse events that may be a consequence of the intervention been reported?	0
9. Have the characteristics of patients lost to follow-up been described?	1

10. Have actual probability values been reported for the main outcomes except where the probability value is less than 0.001?	0
External validity	
11. Were the subjects asked to participate in the study representative of the entire sample from which they were recruited?	1
12. Were those subjects who were prepared to participated representative of the entire population from which they were recruited?	0
13. Were the staff, places, and facilities where the patients were treated, representative of the treatment the majority of patients receive?	1
Study bias	
14. Was an attempt made to blind study subjects to the intervention they received?	0
15. Was an attempt made to blind those measuring the main outcomes of the intervention?	0
16. If any of the results of the study were based on "data dredging", was this made clear?	0
17. In trials and cohort studies, do the analyses adjust for different lengths of follow-up of patients, or in case-control studies, is the time period between the intervention and outcome the same for cases and controls?	1
18. Were the statistical tests used to assess the main outcomes appropriate?	1
19. Was compliance with the intervention/s reliable?	0
20. Were the main outcome measures used accurate (valid and reliable?	0
Confounding (selection bias)	
21. Were the patients in different intervention groups (trials and cohort studies) or were the cases and controls (case-control studies) recruited from the same population?	1
22. Were study subjects in different intervention groups (trials and cohort studies) or were the cases and controls (case-control studies) recruited over the same time?	1
23. Were study subjects randomized to intervention groups?	0
24. Was the randomized intervention assignment concealed from both patients and health care staff until recruitment was complete and irrevocable?	0
25. Was there adequate adjustment for confounding in the analyses from which the main findings were drawn?	0
26. Were losses of patients to follow-up taken into account?	0
Power	
27. Did the study have sufficient power to detect a clinically important effect where the probability value for a difference being due to chance is < 5%	1
Chelly et al 2014	Score
Reporting	
1. Is the hypothesis/aim/objective of the study clearly described?	1
2. Are the main outcomes to be measured clearly described in the introduction or methods section?	1

3. Are the characteristics of the patients included in the study clearly described?	1
4. Are the intervention of interest clearly described?	1
5. Are the distributions of principal confounder in each group of subjects to be compared clearly described?	1
6. Are the main findings of the study clearly described?	1
7. Does the study provide estimates of the random variability in the data for the main outcomes?	1
8. Have all of the important adverse events that may be a consequence of the intervention been reported?	0
9. Have the characteristics of patients lost to follow-up been described?	1
10. Have actual probability values been reported for the main outcomes except where the probability value is less than 0.001?	0
External validity	
11. Were the subjects asked to participate in the study representative of the entire sample from which they were recruited?	1
12. Were those subjects who were prepared to participated representative of the entire population from which they were recruited?	0
13. Were the staff, places, and facilities where the patients were treated, representative of the treatment the majority of patients receive?	1
Study bias	
14. Was an attempt made to blind study subjects to the intervention they received?	0
15. Was an attempt made to blind those measuring the main outcomes of the intervention?	0
16. If any of the results of the study were based on “data dredging”, was this made clear?	0
17. In trials and cohort studies, do the analyses adjust for different lengths of follow-up of patients, or in case-control studies, is the time period between the intervention and outcome the same for cases and controls?	1
18. Were the statistical tests used to assess the main outcomes appropriate?	1
19. Was compliance with the intervention/s reliable?	0
20. Were the main outcome measures used accurate (valid and reliable?	0
Confounding (selection bias)	
21. Were the patients in different intervention groups (trials and cohort studies) or were the cases and controls (case-control studies) recruited from the same population?	1
22. Were study subjects in different intervention groups (trials and cohort studies) or were the cases and controls (case-control studies) recruited over the same time?	1
23. Were study subjects randomized to intervention groups?	1
24. Was the randomized intervention assignment concealed from both patients and health care staff until recruitment was complete and irrevocable?	0

25. Was there adequate adjustment for confounding in the analyses from which the main findings were drawn?	0
26. Were losses of patients to follow-up taken into account?	0
Power	
27. Did the study have sufficient power to detect a clinically important effect where the probability value for a difference being due to chance is < 5%	1
Raeder et al 2015	Score
Reporting	
1. Is the hypothesis/aim/objective of the study clearly described?	1
2. Are the main outcomes to be measured clearly described in the introduction or methods section?	1
3. Are the characteristics of the patients included in the study clearly described?	1
4. Are the intervention of interest clearly described?	1
5. Are the distributions of principal confounder in each group of subjects to be compared clearly described?	1
6. Are the main findings of the study clearly described?	1
7. Does the study provide estimates of the random variability in the data for the main outcomes?	1
8. Have all of the important adverse events that may be a consequence of the intervention been reported?	0
9. Have the characteristics of patients lost to follow-up been described?	1
10. Have actual probability values been reported for the main outcomes except where the probability value is less than 0.001?	0
External validity	
11. Were the subjects asked to participate in the study representative of the entire sample from which they were recruited?	1
12. Were those subjects who were prepared to participated representative of the entire population from which they were recruited?	0
13. Were the staff, places, and facilities where the patients were treated, representative of the treatment the majority of patients receive?	1
Study bias	
14. Was an attempt made to blind study subjects to the intervention they received?	0
15. Was an attempt made to blind those measuring the main outcomes of the intervention?	0
16. If any of the results of the study were based on "data dredging", was this made clear?	0
17. In trials and cohort studies, do the analyses adjust for different lengths of follow-up of patients, or in case-control studies, is the time period between the intervention and outcome the same for cases and controls?	1
18. Were the statistical tests used to assess the main outcomes appropriate?	1
19. Was compliance with the intervention/s reliable?	0
20. Were the main outcome measures used accurate (valid and	0

reliable?	
Confounding (selection bias)	
21. Were the patients in different intervention groups (trials and cohort studies) or were the cases and controls (case-control studies) recruited from the same population?	1
22. Were study subjects in different intervention groups (trials and cohort studies) or were the cases and controls (case-control studies) recruited over the same time?	1
23. Were study subjects randomized to intervention groups?	0
24. Was the randomized intervention assignment concealed from both patients and health care staff until recruitment was complete and irrevocable?	0
25. Was there adequate adjustment for confounding in the analyses from which the main findings were drawn?	0
26. Were losses of patients to follow-up taken into account?	0
Power	
27. Did the study have sufficient power to detect a clinically important effect where the probability value for a difference being due to chance is < 5%	1
Genevois et al 2014	Score
Reporting	
1. Is the hypothesis/aim/objective of the study clearly described?	1
2. Are the main outcomes to be measured clearly described in the introduction or methods section?	1
3. Are the characteristics of the patients included in the study clearly described?	1
4. Are the intervention of interest clearly described?	1
5. Are the distributions of principal confounder in each group of subjects to be compared clearly described?	1
6. Are the main findings of the study clearly described?	1
7. Does the study provide estimates of the random variability in the data for the main outcomes?	1
8. Have all of the important adverse events that may be a consequence of the intervention been reported?	0
9. Have the characteristics of patients lost to follow-up been described?	1
10. Have actual probability values been reported for the main outcomes except where the probability value is less than 0.001?	0
External validity	
11. Were the subjects asked to participate in the study representative of the entire sample from which they were recruited?	0
12. Were those subjects who were prepared to participated representative of the entire population from which they were recruited?	0
13. Were the staff, places, and facilities where the patients were treated, representative of the treatment the majority of patients receive?	1
Study bias	
14. Was an attempt made to blind study subjects to the intervention they received?	0

15. Was an attempt made to blind those measuring the main outcomes of the intervention?	0
16. If any of the results of the study were based on “data dredging”, was this made clear?	0
17. In trials and cohort studies, do the analyses adjust for different lengths of follow-up of patients, or in case-control studies, is the time period between the intervention and outcome the same for cases and controls?	1
18. Were the statistical tests used to assess the main outcomes appropriate?	1
19. Was compliance with the intervention/s reliable?	0
20. Were the main outcome measures used accurate (valid and reliable?	0
Confounding (selection bias)	
21. Were the patients in different intervention groups (trials and cohort studies) or were the cases and controls (case-control studies) recruited from the same population?	1
22. Were study subjects in different intervention groups (trials and cohort studies) or were the cases and controls (case-control studies) recruited over the same time?	1
23. Were study subjects randomized to intervention groups?	1
24. Was the randomized intervention assignment concealed from both patients and health care staff until recruitment was complete and irrevocable?	0
25. Was there adequate adjustment for confounding in the analyses from which the main findings were drawn?	0
26. Were losses of patients to follow-up taken into account?	0
Power	
27. Did the study have sufficient power to detect a clinically important effect where the probability value for a difference being due to chance is < 5%	1
Granados et al 2007	Score
Reporting	
1. Is the hypothesis/aim/objective of the study clearly described?	1
2. Are the main outcomes to be measured clearly described in the introduction or methods section?	1
3. Are the characteristics of the patients included in the study clearly described?	1
4. Are the intervention of interest clearly described?	1
5. Are the distributions of principal confounder in each group of subjects to be compared clearly described?	1
6. Are the main findings of the study clearly described?	1
7. Does the study provide estimates of the random variability in the data for the main outcomes?	1
8. Have all of the important adverse events that may be a consequence of the intervention been reported?	0
9. Have the characteristics of patients lost to follow-up been described?	1
10. Have actual probability values been reported for the main outcomes except where the probability value is less than 0.001?	0

External validity	
11. Were the subjects asked to participate in the study representative of the entire sample from which they were recruited?	1
12. Were those subjects who were prepared to participated representative of the entire population from which they were recruited?	0
13. Were the staff, places, and facilities where the patients were treated, representative of the treatment the majority of patients receive?	1
Study bias	
14. Was an attempt made to blind study subjects to the intervention they received?	0
15. Was an attempt made to blind those measuring the main outcomes of the intervention?	0
16. If any of the results of the study were based on "data dredging", was this made clear?	0
17. In trials and cohort studies, do the analyses adjust for different lengths of follow-up of patients, or in case-control studies, is the time period between the intervention and outcome the same for cases and controls?	1
18. Were the statistical tests used to assess the main outcomes appropriate?	1
19. Was compliance with the intervention/s reliable?	0
20. Were the main outcome measures used accurate (valid and reliable?	0
Confounding (selection bias)	
21. Were the patients in different intervention groups (trials and cohort studies) or were the cases and controls (case-control studies) recruited from the same population?	1
22. Were study subjects in different intervention groups (trials and cohort studies) or were the cases and controls (case-control studies) recruited over the same time?	1
23. Were study subjects randomized to intervention groups?	1
24. Was the randomized intervention assignment concealed from both patients and health care staff until recruitment was complete and irrevocable?	0
25. Was there adequate adjustment for confounding in the analyses from which the main findings were drawn?	0
26. Were losses of patients to follow-up taken into account?	0
Power	
27. Did the study have sufficient power to detect a clinically important effect where the probability value for a difference being due to chance is < 5%	1
Toumi et al 2004	Score
Reporting	
1. Is the hypothesis/aim/objective of the study clearly described?	1
2. Are the main outcomes to be measured clearly described in the introduction or methods section?	1
3. Are the characteristics of the patients included in the study clearly described?	1

4. Are the intervention of interest clearly described?	1
5. Are the distributions of principal confounder in each group of subjects to be compared clearly described?	1
6. Are the main findings of the study clearly described?	1
7. Does the study provide estimates of the random variability in the data for the main outcomes?	1
8. Have all of the important adverse events that may be a consequence of the intervention been reported?	0
9. Have the characteristics of patients lost to follow-up been described?	1
10. Have actual probability values been reported for the main outcomes except where the probability value is less than 0.001?	0
External validity	
11. Were the subjects asked to participate in the study representative of the entire sample from which they were recruited?	1
12. Were those subjects who were prepared to participated representative of the entire population from which they were recruited?	0
13. Were the staff, places, and facilities where the patients were treated, representative of the treatment the majority of patients receive?	1
Study bias	
14. Was an attempt made to blind study subjects to the intervention they received?	0
15. Was an attempt made to blind those measuring the main outcomes of the intervention?	0
16. If any of the results of the study were based on "data dredging", was this made clear?	0
17. In trials and cohort studies, do the analyses adjust for different lengths of follow-up of patients, or in case-control studies, is the time period between the intervention and outcome the same for cases and controls?	1
18. Were the statistical tests used to assess the main outcomes appropriate?	1
19. Was compliance with the intervention/s reliable?	0
20. Were the main outcome measures used accurate (valid and reliable?	0
Confounding (selection bias)	
21. Were the patients in different intervention groups (trials and cohort studies) or were the cases and controls (case-control studies) recruited from the same population?	1
22. Were study subjects in different intervention groups (trials and cohort studies) or were the cases and controls (case-control studies) recruited over the same time?	1
23. Were study subjects randomized to intervention groups?	1
24. Was the randomized intervention assignment concealed from both patients and health care staff until recruitment was complete and irrevocable?	0
25. Was there adequate adjustment for confounding in the analyses from which the main findings were drawn?	0

26. Were losses of patients to follow-up taken into account?	0
Power	
27. Did the study have sufficient power to detect a clinically important effect where the probability value for a difference being due to chance is < 5%	1
Muijen et al 1991	Score
Reporting	
1. Is the hypothesis/aim/objective of the study clearly described?	1
2. Are the main outcomes to be measured clearly described in the introduction or methods section?	1
3. Are the characteristics of the patients included in the study clearly described?	1
4. Are the intervention of interest clearly described?	1
5. Are the distributions of principal confounder in each group of subjects to be compared clearly described?	1
6. Are the main findings of the study clearly described?	1
7. Does the study provide estimates of the random variability in the data for the main outcomes?	1
8. Have all of the important adverse events that may be a consequence of the intervention been reported?	0
9. Have the characteristics of patients lost to follow-up been described?	1
10. Have actual probability values been reported for the main outcomes except where the probability value is less than 0.001?	0
External validity	
11. Were the subjects asked to participate in the study representative of the entire sample from which they were recruited?	1
12. Were those subjects who were prepared to participated representative of the entire population from which they were recruited?	0
13. Were the staff, places, and facilities where the patients were treated, representative of the treatment the majority of patients receive?	1
Study bias	
14. Was an attempt made to blind study subjects to the intervention they received?	0
15. Was an attempt made to blind those measuring the main outcomes of the intervention?	0
16. If any of the results of the study were based on "data dredging", was this made clear?	0
17. In trials and cohort studies, do the analyses adjust for different lengths of follow-up of patients, or in case-control studies, is the time period between the intervention and outcome the same for cases and controls?	1
18. Were the statistical tests used to assess the main outcomes appropriate?	1
19. Was compliance with the intervention/s reliable?	0
20. Were the main outcome measures used accurate (valid and reliable?	0
Confounding (selection bias)	

21. Were the patients in different intervention groups (trials and cohort studies) or were the cases and controls (case-control studies) recruited from the same population?	1
22. Were study subjects in different intervention groups (trials and cohort studies) or were the cases and controls (case-control studies) recruited over the same time?	1
23. Were study subjects randomized to intervention groups?	1
24. Was the randomized intervention assignment concealed from both patients and health care staff until recruitment was complete and irrevocable?	0
25. Was there adequate adjustment for confounding in the analyses from which the main findings were drawn?	0
26. Were losses of patients to follow-up taken into account?	0
Power	
27. Did the study have sufficient power to detect a clinically important effect where the probability value for a difference being due to chance is < 5%	1
